# Learn, Have Fun and Be Healthy! An Interview Study of Swedish Teenagers’ Views of Participation in Club Sport

**DOI:** 10.3390/ijerph18136852

**Published:** 2021-06-25

**Authors:** Britta Thedin Jakobsson, Suzanne Lundvall

**Affiliations:** 1 The Swedish School of Sport and Health Sciences (GIH), 114 86 Stockholm, Sweden; suzanne.lundvall@gih.se; 2 Department of Food and Nutrition, and Sport Science, University of Gothenburg, 411 20 Gothenburg, Sweden

**Keywords:** club sport, health promotion, salutogenic, teenagers, young people

## Abstract

In Sweden, participation in club sport is a vital part of many children’s lives. Despite this, many stop in their teenage years, raising questions concerning if and in what ways club sport can provide health-promoting activities via longer, sustained participation. The aim of this cross-sectional study is to explore and discuss young people’s views of club sport from a health-promoting perspective. The analysis draws on three sets of qualitative data: results from focus groups interviews conducted in 2007 (*n* = 14) and in 2016 (*n* = 8) as well as 18 in-depth interviews conducted 2008. Antonovsy’s salutogenic theory and his sense of coherence (SOC) model inspired the analysis. Teenagers want to be a part of club sport because of a sense of enjoyment, learning, belonging and feeling healthy. Teenagers stop when sport becomes too serious, non-flexible, time-consuming and too competitive. The urge for flexibility and possibilities to make individual decisions were emphasised in 2016. The organisation of club sport, it seems, has not adapted to changes in society and a generation of teenagers’ health interests. Club sport has the potential to be a health-promoting arena, but the focus should be on changing the club sport environment, instead of a focus on changing young people.

## 1. Introduction

Many young people engage in sport because it is fun and joyful [1,2]. In Sweden over 80% of young people participate in sports clubs under the umbrella of the Swedish Sport Confederation (SSC) at some time during childhood [3,4,5]. Different factors, such as social class [6,7,8,9,10], gender [11,12], and parents’ own interest in sport [11,13], affect teenagers’ participation and continuing in club sport. Studies have shown that children who participate in organised sport are more physically active than those who do not [14,15,16], and that in their teenage years club sport participants see themselves as more physically active than non-participants [17]. Furthermore, children are more active on days involving organised sport than on non-sport days [9]. During the last 10 years in Sweden, the number of dropouts from club sport among children and adolescents have increased [3,18] and the Swedish government has allocated additional money to local sports clubs belonging to the SSC in order to stimulate more young people to be physically active by participating in club sport [19,20].

While youth sport primarily takes place in school in countries such as the USA, Canada and the UK, voluntary sports clubs play an important role in this respect in the Scandinavian countries [21,22]. In Sweden, organised club sport has been part of the development of the welfare state and policy since the beginning of the 20th century. There is a consensus between the state and the SSC that the Swedish sports movement’s role is to support democracy, equality and public health [19]. The Swedish State and the SSC share a mutual, if implicit contract [19,21,22] with the joint goal that children, adolescents and adults should be encouraged and given opportunities to play sports and take part in exercise, based on their own interests and circumstances [19,23]. The SSC’s policy document [23] emphasises that the sports movement shall be open for all, regardless of whether their ambitions are competitive or purely recreational. Activities should be adapted to young people’s varying levels of ambitions, and participation in both recreational and elite sports is to be encouraged. Over the past decade, researchers have paid interest to how youth sport can be seen as sites for health promotion and have highlighted the potential of sports clubs to become health-promoting settings when participants are doing their core sporting activity [24,25,26,27]. Club sport can accordingly reach large groups of people and increase the physical, psychological and mental health of their participants through sport and physical activity practice [28,29,30,31]. Furthermore, Kooko [32] has formulated guidelines for clubs to enhance health promotion as a part of their activities. Geidne and colleges [27] reformulated previous guidelines when implementing a setting-based health promotion approach. However, there is still a lack of formal guidelines for health promotion in sports clubs belonging to the SSC. Although club sport may promote physical activity, physical activity does not necessarily promote health [32]. Since the sports movement is an arena which many young people come into contact with, it is important to find out more about what young people value and what enhances their feelings of wellbeing in club sport participation.

Despite a good body of work on health promotion and club sport, knowledge is still lacking as to how club sport can be an attractive arena for health promotion attractive for young people to engage in. Thus, the aim of this, qualitative cross-sectional study is to explore young people’s views of club sport from a health promoting perspective. This is done by analysing results from semi-structured focus group interviews with students from the same schools conducted in 2007 and 2016 and in-depth interviews with club sport participants conducted in 2008. The research questions are:

- What in club sport participation creates feelings of well-being and/or unwell-being amongst young people?
- What are the similarities and differences in the different datasets concerning views of club sport participation?
- Which aspects of club sport, as described by young people, provide a framework for health promotion?

The results and possible implications are discussed in relation to club sport as a health-promoting arena and what may support young people to participate and continue with club sport. This is in line with research questions posed by sociologist Aaron Antonovsky and the salutogenic theory [33,34] dealing with what creates sense of coherence (SOC). Antonovsky’s [33,34] salutogenic theory and SOC model, with the components of comprehensibility, manageability and meaningfulness, have served as analytical tools in the study. A salutogenic perspective can also be seen in relation to health promoting practices [35] that sports clubs can adopt.

## 2. Materials and Methods 

In this qualitative interview study the empirical data that form the base for this article comes from two research projects; the longitudinal and multidisciplinary research project, *School-Sport-Health* (SSH) [36], and the project *Who stays and why—a study of young people’s participation in club sport* funded by the SSC [37]. These two projects provided the opportunity to address the above named research questions.

The SSH project initiated a base study in 2001 with 48 randomly selected Swedish schools. In total 1976 students aged 9, 12 and 15 (grades 3, 7 and 9) participated [30]. Research questions included studies of sport participation, self-reported physical activity, participation in physical education, as well as studies of physical status and motor competence. The two datasets of focus group interviews were conducted in 2007 (the SSH 2007 study) with grade nine students from seven schools and in 2016 (the SSH-2016) with four schools from the original base study [36]. The third data set consists of the in-depth interviews in 2008 with teenagers participating in club sport [38]. Some of the empirical material has been used in other studies [39,40], but with different aims and research questions.

### 2.1. Participants

In the SSH-2007 study, 14 focus semi-structured group interviews were performed with 66 school year nine students (31 girls and 35 boys aged 15). In the SSH-2016 study eight corresponding focus group interviews (30 boys, 18 girls aged 15) were conducted. The SSH-2007 participants were selected based on a strategic sample of seven schools and the SSH-2016 of four schools that had participated in the SSH-2001 baseline study [36]. As in the baseline study, the samples took a geographical spread of schools across the country and the size of the municipalities into account (Table 1). For both of these two studies we asked the physical education teachers in the selected schools to request volunteers that would like to participate and who were either active or non-active in club sport. Thereafter the teacher considered which students had expressed an interest in participating and then made the final selection.

The participants in the third data set from the study *Who stays on and why* from 2008, with 18 in-depth interviews were conducted with nine girls and nine boys (aged 15–19) participating in eight different sports (athletics, basketball, equestrian sports, floorball, football, handball, swimming and ultimate frisbee). The selection of sports and sports clubs was made using SSC’s database covering, at that time, its 69 individual sports federations [38]. A target sample selection was carried out to find young people who were not among the selected sports’ elite. To obtain information-rich cases [41], clubs were contacted and their trainers provided us with the names of teenagers who were suited to the research aim and questions. The teenagers were then contacted individually by e-mail and telephone.

### 2.2. Interview Procedure

One of the authors of this article collected the data from the studies performed in 2007 and 2008, and the data from the study in 2016 were collected by both authors. The focus group interviews and the 18 in-depth interviews were conducted in a similar way: 60–90-min in length and digitally recorded in the form of a private conversation to encourage openness and follow-up questions. A core interview guide for the focus group interviews and one for the club sport participant in-depth interviews were used with semi-structured questions based on the previous literature [41,42]. The purpose was to combine an exploration of several subject areas with a certain amount of freedom in the order and scope of the questions [42]. The focus group interviews questions were about leisure time practices, including club sport and the school subject of physical education and health. The in-depth interviews began with questions about how the teenagers would describe themselves. Thereafter, the questions focused on the teenagers’ reasons for continuing with sport and the factors that made it easy or difficult to continue. An inductive qualitative content analysis guided the procedure for analysing the three sets of empirical material. Two pilot interviews were conducted in 2007, 2008 and 2016 to gain familiarity with the interview guide.

### 2.3. Ethical Considerations

All three interview studies were carried out in accordance with ethical guidelines [42,43], including gathering informed consent from all the participants and the parents of students under 15. At the outset of each interview, the respondents were guaranteed confidentiality and told that they were not obliged to answer the questions and could leave the interview at any time.

As the data are treated as confidential, we do not identify the participants by their real names, school or sport club, nor do we reveal other easily traceable information in the presented results. We obtained consent for each school’s participation from the principals and the parents of the teenagers involved in the in-depth interviews. Students from the selected classes received information about the study in a PE lesson. The parents of students who had expressed interest in the study received information about it and together with the student agreed on participation [43]. None of the students or club sport participants withdrew from the studies.

### 2.4. Transcription and Analysis with a Salutogenic Approach

The purpose of a qualitative interview is usually described as trying to understand the respondents’ world [41]. The method used and the questions posed raise theoretical questions about how the respondents describe their situation to someone else (the researcher), in this case about the teenagers’ leisure time, participation in sport in clubs and at school. What is talked about in the interviews is then analysed in terms of what can be seen as meaningful and important with regard to participation (or not) in club sport. The respondents’ opinions about and experiences of sport, what they have to deal with, comprehend and find meaningful, tell us something about their participation in and experience of club sport.

This is in line with Antonovsky’s salutogenic approach [33,34] when exploring why people stay healthy [33]. Antonovsky concentrated on the processes and resources for health and not the risks of disease [33,34]. His SOC model with the components, comprehensibility, manageability and meaningfulness was developed to analyse what these health resources might consist of. He designed a survey based on these components and used both questioners and interviews in his research projects [34] (Appendix) He claimed that the ability to comprehend someone’s situation in life and the capacity to use resources explained why people in stressful situations managed to stay well and, in some cases, even improved their health. The salutogenic perspective and SOC model inspired us when analysing the interviews and discussing club sport from a health promotion perspective. The fact that the study is inspired by Antonovsky means that we have taken the liberty of interpreting and using his concept when formulating the research questions and analysing the interviews. If young people consider their club sport participation to be comprehensible (they understand the logic of sport), manageable (they can do what is required and accept the conditions and rules) and meaningful (they are motivated and like what they are doing), they are more likely to continue with it [39]. In other words, the assumption is that SOC is needed if youth are to remain in club sport.

Comprehensibility, one of the components of the SOC model, is the extent to which events are perceived as logical, ordered, consistent and structured. Comprehensibility is experiencing life in all its forms as real, tangible and structured, rather than random and inexplicable. The second element, manageability, is the extent to which a person feels that they have the resources to meet the demands made and the challenges set. Antonovsky’s third element of SOC is meaningfulness: the extent to which we feel that life makes sense and that challenges are worthy of commitment whether or not we have positive expectations of life and the future, and feel that situations in life are challenging, interesting and worthy of emotional commitment. However, as Antonovsky also puts it, salutogenesis and pathogenesis are complementary [34] and can be seen as two sides of the same coin. Therefore, using the salutogenic approach also indicates when there is a low feeling of comprehensibility, manageability and meaningfulness [34] (Appendix). Antonovsky’s salutogenic theory and the SOC model have been used in Swedish as well as international research on health and resilience [44,45], health and learning in education, and especially in the subject physical education [35,46,47,48,49] and health-promotion work with teenagers [50,51].

The analysis began with the writing of field notes immediately after the interviews, listening to the recordings and then reading the transcripts word for word several times. During the listening and reading, notes were made in the text, especially regarding the question: what does this statement say about experiences of club sport participation, feelings of well-being and club sport as a health promoting activity? After reviewing the transcripts, the initial notes were transformed into concise phrases that captured the essence of what was reported. Next, the transcripts were coded into meaning units from each piece of text considered relevant to the study’s aim [41]. The core comments were categorised into sub-themes and themes [41]. This part was largely inductive [41]. After the first categorisation of empirical material into meaning units and themes, the analytical procedure continued by paying attention to the three components in Antonovsky’s SOC model, namely to what emerged as comprehensive, manageable and meaningful or not in relation to the sport participation, health and wellbeing in the themes appearing in the interviews. We critically discussed two interviews from each data set, the coding density and the relevance of each meaning unit to the study’s aim and research questions. Next, the other 16 in-depth interviews from 2008 and the 22 focus group interviews from 2007 and 2016 were coded separately and the degree of coherence examined. After that, we compared the categories of themes we had each found. While these essentially corresponded to each other, we discussed what the theme should be called before reaching an agreement.

In the empirical material in all three data sets, the respondents highlighted ‘people do sport because it’s fun’. As our intention was to gain a deeper understanding of differences and similarities in the data sets of ‘what makes teenagers participate or not in sports’, and their responses whether it is either fun or not fun, we decided to explore this more in depth. Using SOC’s analytical tools, several aspects of fun emerged that we consider to be closely related to meaningfulness and that contribute to SOC in the respondents’ lives [33,34,44,50,51]. More precisely, expressions associated with meaningfulness, such as participation, involvement, enjoyment, solidarity, interest, motivation and belief in the future, were utilised in the analysis [33,34,50,51]. We assumed that those who remained active in club sports found sport comprehensible, manageable and meaningful. If not, they would probably have stopped being involved. By exploring the SOC components our ambition was to gain a greater understanding of the well-being and health promoting perspectives on club sport participation by clarifying what seemed comprehensible, manageable and meaningful or not about participation.

## 3. Results

Three main themes and two sub-themes from the analysis of the interviews were identified as: (1) sense of learning and developing, with two sub-themes: ‘competing as a challenge and fight for the moment’, and ‘coaches make sport manageable, comprehensible and meaningful… or not’, (2) sense of belonging and being part of a group, and (3) sense of feeling healthy.

### 3.1. Sense of Learning and Developing in Club Sport

In all three data sets of the empirical material, learning and developing sporting skills and feelings for the game were mentioned in relation to having fun and enjoying sport. Johan said: ‘You play football because it’s fun, that’s the whole idea with football … You want to learn and develop’ (2008, Johan). This was a common response among the respondents. Carol, from one of the focus group interviews, said:

To learn a new dance, to learn something new … so you learn so much about pace and melodies and so … control your body better and feel like a pro … you move like a professional, you can control and rule yourself. You develop every time you practice … it feels so good … it’s so much fun

(2007 B6 Carol).

Learning something new can also mean succeeding with something after practising it for a long time and understanding a tactical movement better in a game. According to the respondents, meaningfulness in participation seemed to improve by developing and being able to handle new situations, as well as gaining a deeper understanding of a specific sport. Being able to master motor skills, be in physical shape, understand what the game was about, do what was required and be in control of yourself were emphasised on numerous occasions in the interviews. In order to have fun in sport, young people need to understand what to do, are willing to practice hard and meet the requirements of club sport. Antonovsky includes these aspects in his concepts of comprehensibility and manageability, which mean that teenagers have to be able to continue even if they sometimes find the sporting activities tedious and monotonous. Daniel said:

I thought it [basketball] was boring at first but I managed to keep fighting and didn’t give up. The best players were my role models. Improve myself. It is a fantastic feeling when everything goes right

(2008 Daniel).

The young people often expressed the concepts of learning and development in terms of feelings and experiences, for example ‘the wonderful feeling’ they experienced ‘when everything is right’. Some described being exhausted when practising or that when getting everything right it felt enjoyable and satisfying. They also expressed unpleasant feelings, such as being ‘ready to vomit’ or being ‘brutally’ tired. They often talked about how wonderful it felt after the workout. For them, sport become a tangible physical experience with contrasting feelings of pleasure and discomfort.

Motivation, determination and finding sport manageable and comprehensible is necessary in order to continue with it, even if it is sometimes difficult, tiresome and unexciting. Among those who had participated in club sport but had now stopped, a common expression was ‘sport practice became boring’ (2016 B1 Kate). Joe said:

At first it was fun but then it got boring. That was when you had to do the same thing over and over again and just improve on the basics, it was boring. Everyone already knew everything, and you couldn’t move on

(2007, C1 Joe).

Another expression was ‘It became demanding to go and practise. You had to be so skilled’ (2016 B2 Pia). For those who had left sport, the comprehensibility and manageability had seemingly disappeared with the lack of new challenges and the monotonous nature of sport, and when motivation and enjoyment diminished or ceased. For many of the participants, the opportunities for learning seemed to be more important than the opportunities to compete. The respondents seemed to be mainly interested in a sport logic that included learning physical skills.

Another thing that was mentioned in the 2016 interviews but not in 2007 and 2008 was watching films on social media of sport and then trying to do the same movements themselves. Claes said that: ‘I watch these parkour tricks and then try to do them myself’ (2016, B2 Claes). Others talked about watching football tricks or dance moves that they then tried to mimic. Also, some of the respondents had left club sport and did for example skateboard and snowboard as self-organised sport [52,53]. Another difference that emerged in the interviews was that the 2007 respondents consumed football as spectators on site. Per said: ‘I love to see and cheer for my team’ (2007, A1, Per). In 2016, several of the respondents mentioned that they played world-wide tournaments on the internet: ‘I play FIFA [a football computer game] with people from all over the world’ (2016 B1 Jesper). While the opportunities of learning and development through social media have increased, several aspects of participating in club sports in real life seem to be challenged.

The respondents often talked about the importance of feeling that they had mastered a sport and the training elements that accompanied it. When contrasting the answers in the interviews, those who no longer participated in sport wanted more flexibility and opportunities to decide for themselves what to practise and when. This was mentioned more often in 2016 than in 2007 and 2008. A common answer was the lack of time due to schoolwork, that ‘school takes up too much time and if you miss a practice you can’t continue’ (2016 D2, Sara). Also, expressions like Ricky’s (2016 B1) ‘you need to study a lot in school to get high grades so you have to spend less time to practicing football’. In other words, an important aspect was being physically competent enough to cope with the demands of sport, which increased with age. It was both about training more and having a greater physical and athletic ability with more complexity, such as motor skills and game perception. But it was also about being able to plan, prioritise and negotiate their time between sporting activities and schoolwork. This was more often expressed in the interviews in 2016 as well as the importance of deciding, controlling and governing yourself.

Several of the respondents described sport as a sensual experience of learning and development that they would not want to be without. Edgar, had been skating and practising athletics and floorball for several years said: ‘I don’t know if I could live without sport’ (2016, B1, Edgar). For Edgar, sport had become a lifestyle and was meaningful in the sense of being able to learn something new and have new challenges and experiences. ‘The meaningfulness’ of sport seems to be found in the bodily experience and as an intrinsic value; a feeling of being here and now rather than a cognitive thought. This feeling was important to the respondents who invested a lot of time and energy in their sport and felt committed to it.

#### 3.1.1. Competing as a Challenge and Fight for the Moment

The respondents expressed enjoyment, learning and development in connection with competitions and games. Erika said she loved ‘to compete, play games and matches because it’s exciting’ (2016, A2 Erika). Although the respondents talked about competing, competitions and matches, very few mentioned competing as an important reason for participating in club sport. Only a few stressed that competing was important and the reason why they continued to take part in sport. For Eva, it was the fight for the moment that was important and gave spirit to the game:

Sure, you can practise and get fit by doing gym or fitness training, but practising floorball is different. There is action and nerve when there’s matches. It is exciting and it makes me do my best. Afterwards I forget who won. That’s not important

(2007, C2 Eva).

Being involved in competitions was an important experience—more so than winning. One interpretation is that competitions and games may not be the primary reasons for continuing with sport. Another interpretation is that sport means competition and matches. Those who were no longer involved in club sport often said that they stopped because they did not want to compete. ‘You had to participate in competitions if you wanted to be in the club. I am not a person who likes to compete, that is why I stopped’ (2016, C2 Paul).

Even if some only wanted to participate at a recreational level, such as once or twice a week and not engage in games and competitions, they were nevertheless obliged to compete often and practise several times a week. Club sport for recreational reasons seemed to involve competition, which could make participation difficult or impossible for some.

When talking about competitions in the interviews, the respondents often described competitions and matches as motivating, opportunities to acquire new skills and a common goal that united the team. They did not primarily express an interest in competing at an elite level, although this could be a driving force for some. On the other hand, they often returned to the importance of understanding and being able to deal with the competitive elements of sport in order to continue.

#### 3.1.2. Coaches Make Sport Manageable, Comprehensible and Meaningful … or not

A recurring theme in the interviews was the desire and importance of educated, experienced and committed leaders. Those who had continued with sport often mentioned coaches who made them come to training sessions. Those who had stopped doing sport expressed their opinions about the lack of good and competent coaches: ‘The coach should be a nice person, a person who wants you to do your best. But it is not enough to be kind. They have to be competent as well’ (2016 D1, Caroline). Some had changed to another sport because they did not like the coaches. Bella (2007 B5) said that she changed because she could not stand the bullying from the coaches. Bengt said:

I also played ice hockey but quit a few years ago. I played because it was fun to play with the team. But after a while it got competitive and serious, and the coach was not good. I wish we’d had coaches who helped us to develop our game and skills

(2016, C2 Bengt).

In another interview, Oba said:

I had a coach who always made you feel welcome. He was serious about what we were supposed to learn and practise. It turned out that he was interested in us as people, not just as players

(2007 A2, Oba).

Others stated that some coaches never said anything positive but were just demanding. ‘They could be very good at handball, but they were always nagging and favoured some’ (2016 A1, Aron). Well-educated coaches played an important role in making sport manageable and comprehensible. Without engaged and educated coaches who showed interest in the respondents, it was unlikely that club sport would become a health-promoting activity.

### 3.2. The Sense of Belonging and Being a Part of a Group in Club Sport

In the interviews the respondents returned to the joy and importance of feeling fellowship, community and solidarity with other participants and coaches, as it was these aspects that made sport fun and meaningful for them [2,35,41,42,43,44,45,46,47,48,53,54,55]. Some of those who had stopped playing sport emphasised that they wanted to start again but that their friends did not want to join them. They emphasised that it took courage and self-esteem to dare to go alone to a new sports club. Cane said:

It was the community itself that was the reason I went there. When I realised that it was not like that any longer it was no longer fun. If I find someone who wants to join me I might start again. I don’t want to go alone

(2007, D1 Cane)

Some of those who had stopped being involved in club sport had started again because they missed doing sport together with friends: ‘I started again because I missed my sport mates and the sporting community too much’ (2007, A2 Janne), and ‘You want to get a break from home, meet others and do something fun together’ (2016 A1, Hanna). Later in the interview, Hanna said: ‘You learn to socialise by playing sport’. She believed that sport differed from other activities, such as social media on the internet. For her, having a specific task to solve in the game itself or in the training sessions, combined with being able to mix seriousness, concentration, fun and enjoyment, made sport a special phenomenon. Friendship in teams and training groups was an important reason (for some the most important) to engage in sport. However, bullying and exclusion were mentioned as reasons why some of the respondents had stopped doing sport, even if only temporarily. In the focus group interviews the respondents talked about a coach who bullied some of the players to the extent that they stopped playing sport because they were unable to accept that kind of leadership (2016, B2). During both the focus and in-depth interviews, the respondents emphasised the importance of everyone being able to participate and make their voices heard in sport so that it did not lose its meaning [4,56]. Despite the difficulties that some of the respondents encountered in clubs, sport seemed too important and meaningful to relinquish.

Abbot: It’s fun and so ... I feel good about training and meeting friends. Otherwise, I become lazy and passive.

Interviewer: But couldn’t you meet friends in other activities, like playing computer games together? Why do sport?

Abbot: Oh… you are together and socialise without talking.

Tom: Yes, you are physical and interact and share experiences by practising

(2007 D2 Abbot and Tom).

Sport can thus contribute to meaning-making, provide opportunities to socialise and feeling part of a community without the need for conversation and discussion.

There were statements about not really having time to train or feeling uncomfortable or worried about it. Despite this they still went to training sessions because they did not want to disappoint anyone, that sport seemed meaningful to them, and that they did not want to be without it. Helle said:

The importance of good friends and coaches is priceless. You can feel that things have gone badly during the day [at school] and then you come to training sessions and do something together and they are happy to see me. It is such a good feeling

(2007, A2 Helle).

### 3.3. The Sense of Feeling Healthy

Some of the statements in the focus group interviews indicated that staying physically fit and with feelings of wellbeing were important reasons for engaging in sport. This included improving cardiovascular conditioning, preventing disease or illness, sharpening their concentration, building up strength, or just being able to relax and cope with life. Some respondents stressed that sport and fitness training made them look good. Especially those who were no longer involved in club sport but continued at commercial gyms or fitness clubs talked about physical activity in terms of improving their appearance. ‘I do fitness training because I am not satisfied with my body’ (2007, A2 Alex), or ‘I want to lose weight’ (2016 D2, Caro). Obviously, some had an interest in working out in order to keep in shape and improve their looks:

Strength training is tough, it’s no fun, you stand there and do some exercises. My brother said that after a month then your muscles get bigger. Before that it’s just shit, nothing happens, it’s just hard. Now I’m doing it for preventative reasons but it’s not fun

(2016, B2 Carl).

Those who took part in club sport often focused on health from a more holistic perspective and had ‘a good feeling in both body and soul while doing sport’ (2008, Tove). They emphasised that they felt better when they were physically active and that sport helped them to be active. Other words used to describe sport and physical activity were ‘wholesome’ and ‘salutary’, which is similar to Eriksson’s [44] suggested salutogenic approach to physical activity as a health-promotion arena. Ebba said that: ‘Sport makes me relax, it helps me to focus and switch off from other things’ (2016, D1 Ebba), while Anders said: ‘You feel good about yourself … You get more self-confidence when playing sport’ (2007, D4 Anders). International studies have also revealed that young people engage in sport because it makes them feel healthy [1,2,54,55]. Sport helped some of the respondents to relax from school. Marcus said: ‘You think mostly about what you do there and then [at sport practise]’ (2007, B1 Marcus). Harald continued, ‘You think that you should do your best in the training session [basket]. You can’t think of school at the same time‘ (2007, B1 Marcus, Harald). Sport also helped the respondents to organise their lives and made them feel good about themselves. Eve said:

So you feel good about it … so I feel good about playing sport … so I cannot understand why people stop. Now that I play sport, I really have time for everything, I am efficient and organised

(2008 Eve).

However, for others sport was too time consuming and was often why they stopped. Especially in the interviews in 2016, many of the respondents talked about the pressures at school and that this made club sport sessions too much for them to handle: ‘It also takes too much time to prepare for competitions, so that’s why I stopped’ (2016 D2 Folke).

For some of the respondents club sport participation gave them a sense of well-being since they could relax, being a tomboy, being playful, not having to think about appearance and just doing sport for enjoyment. Kim said that ‘I can be aggressive, show all my feelings and don’t have to put on make-up when doing sport’ (2007 A2 Kim). Ove continued by saying that: ‘It’s hard to know why you continue ... maybe it’s because you develop as a human being and have a good feeling of well-being’ (2007 A2 Ove).

What was perceived as comprehensible, manageable, meaningful and important for the respondents was the ability to learn sporting skills, experience development, overcome challenges and make friends. For some, club sport was something that made them feel healthy and able to deal with everyday life. For others it was something that they wanted to do but that they did not have the right skills or enough time for, or that no sport club could support their ambitions. For some, club sport participation enabled them to experience well-being and SOC in the moment.

## 4. Discussion

This qualitative cross-sectional study of focus group interviews conducted in 2007 and 2016 and in-depth interviews conducted in 2008 is limited in size and research design, which means that no general conclusions can be drawn. However, it is not our intention to generalise the findings from this qualitative study. Nevertheless, the salutogenic-inspired approach helps us to understand something about how young people view and reason about club sport participation and the environment surrounding this practice. Our reading of their experiences of participating and not participating in club sport says something about club sport as a possible arena for health promotion. The latter is particularly relevant in light of recent efforts in Western societies to increase physical activity for children and adolescents [18,19,57,58].

One key result is that even though the interviews were conducted almost 10 years apart, several of the findings are similar. In the analysis and discussion inspired by salutogenic theory with the SOC model a main finding is that club sport participation still emerges as fun and enjoyable in terms of learning and development. In the teenage years, it is more common in club sport to emphasise sport participation in an elite context. Therefore it is somewhat surprising that teenagers stress the importance of learning and development when participating. According to Côté and Hay [2], one would have expected them at this age to have emphasised the importance of competing and the ambition to become the best (see also Kirk [58]). These statements are both from those who are still involved in club sport and those who are not (see also [59]). Moreover, club sport provides structure in young people’s lives and provides a meaningful and enjoyable context for the moment. This is something that those who no longer participate miss and want to experience again.

Another similar finding in relation to the different datasets is that for the most part the teenagers do not stress winning and the striving for competitive success.

Very few of the respondents reported having elite ambitions for the sport they were engaging in. Instead, they seem to be satisfied with sufficiently difficult challenges that help them to learn and develop. Some of those who had continued with sport found it natural to engage in an activity that included competition, even if they also emphasised that, for them, social interaction and skills development were the most important [24,60].

The importance of having well-educated and thoughtful coaches and leaders who are serious about sport and who pay attention to all individuals is also consistent throughout the empirical material. Eliasson and Jansson [61] has shown that sports leaders are important for children’s experiences of sports, (see also [62,63]). The respondents talked about leaders who have helped them to continue with sport, as well as those who have made them leave. Some of the respondents felt competent and motivated enough to reconnect and find a new sport club or a new sport, while others stopped doing sport altogether.

Although there are more similarities than differences between the empirical studies, some aspects differ. In the 2016 interviews, time and flexibility emerge somewhat more challenging for the respondents, which may be due to changes in society with a stronger focus on individual performance [5,40,64]. In all of the 2016 focus group interviews the respondents talked about the importance of school success, but this is not highlighted in the material from 2007 and 2008 [40,65]. This may be traced back to a passed legislation in 2011, introducing an earlier assessment of knowledge, with grades from school year six instead of school year eight, accompanied by stricter knowledge requirements and assessments in the Swedish school system [66].

Another critical aspect to bear in mind, which becomes visible in 2016, may be the increasing influence of social media in relation to how sport practice is organised and communicated. However, it could also have an inhibitory effect, in the sense of needing to be perfect in order to look healthy, fit in, or do the perfect move. In 2016, club sport emerges as more of an individual investment where young people have to make active choices and take responsibility for them. On the one hand, they express a desire for team spirit, belonging and sharing values with others, while on the other hand they express that they want to or should develop in their own health and wellbeing. It seems to be a balancing act between independent, active decisions and/or trying to fit in with the club sport structure. Similar ways of reasoning are found in Hedenborg and Glaser’s [64] study of young people’s experiences of organised sport and their expectations of future sporting environments.

In the interviews, sport seems to be manageable, comprehensible and meaningful for the respondents still participating, although what is meaningful seems to vary. Sport provides a social cultural space and an environment where teenagers can experience meaningfulness in terms of learning, developing physical ability, experiencing a feeling of belonging, being challenged and feeling heathy. In this way, club sport fills an important function in young people’s lives by making it possible for them to have a physically active lifestyle experiencing SOC [39]. It is conceivable that club sport could make use of a salutogenic approach where learning processes are in focus, in order to strengthen teenagers’ resources and help them to live a good life [35]. This is in line with previous studies of Geidne and colleges [24] suggesting that club sport for young people can act as health-promoting settings. However, club sport practices, aims and purposes, have to incorporate a comprehensive approach where the young athletes and their everyday lives are put at the center. However, questions remains regarding how well club sport can meet these demands. Furthermore, club sport has to compete with a rapidly growing commercial market of light communities for fitness training. New forms of sporting practices, including both the light communities and the self-organised, have affected the sport practices of the respondents in the SSH 2016 [40,52,53,64].

The results indicate, especially in 2016, that in order to participate and experience some sense of wellbeing, young people need to conform to the sport club culture. Additionally they have to accept that organised club sport is not tailored to their different requirements. The results of the study show that both the sport movement and the state need to reconceptualise club sport in order to encourage more young people with different ambition levels to continue as participants. This could be done by focusing on aspects in club sport that provides a framework for health promotion such as young people’s willingness to learn and develop [1,2]. It is central to create an environment where there are opportunities to learn, become physically and socially competent, and to experience a sense of belonging. A thoughtfully and well-conducted sport practice can create resources that underpin experiences of manageability and comprehensibility as well as increase opportunities to participate in physical activity and sport. One of the strengths of club sport is regularity, continuity and a context where teenagers are sought after. Asking questions about what teenagers need to understand, manage and experience as meaningful in sport would enable club sport to re-organise in order to attract more young people to participate and continue to do so longer in life. Furthermore, club sport could incorporate different approaches with more flexibility and possibilities for young people to decide and influence the sport practice.

## 5. Conclusions

This qualitative study shows that young people want to participate in club sport, but on their own terms and conditions. This is more apparent in the more recent interviews in 2016 than in those from 2007 and 2008. Antonovsky [33] argues that it is imperative to advance new conceptualisations of health, particularly in health promotion practices. His concepts were useful when investigating teenagers’ views of club sport and possible resources for health development in club sport. Doing sport seems to be manageable, comprehensible and meaningful, giving a sense of wellbeing for those who continue. However, what is meaningful seems to differ in some aspects. Teenagers are at a critical age of transition, and sport can provide in-person meetings and activities, socialization, and the sharing of experiences together with others, which are important parts in experiencing wellbeing. Maybe changes in society, such as the ubiquity of social media, smartphones, as well as, a new school discourse, not only challenge young people’s possibilities and willingness to participate in club sport, but also future policies and initiatives for organised club sport. Changes in society may also have affected and changed young people’s views and expectations of sport participation. The sports movement does not seem to have kept up with this development and has insufficiently met young people’s varying desires and requirements. An important direction for the organisation of club sport might be to change and adapt to a generation of young people valuing social interaction, interest in health, community and sporting challenges [5]. Instead of a focus on changing young people, the focus should be on changing the club sport environment. With such an approach, club sport has a potential to be and become an attractive arena for health promotion for young people not least in a fragmented and challenging time. A potential direction for future research could be to observe club sport practices in order to gain further insight into young people’s preferences of sport activities, learning, health and well-being, as well as gaining a critical understanding of workings of power, gender and privilege and subordination of youth.

## Figures and Tables

**Table 1:** Overview of schools coded with the year of the study (2007 and 2016) and the letters A–D to provide information about which focus group interview the quotes come from.

2007	Place and School
66 Participants	
14 Focus Group	
3–6 Students/Group	
2007 A1	Big city (800,000 inhabitants) city centre school
2007 A2	Big city (800,000 inhabitants) city centre school
2007 B1	Suburb school of a big city 61,500 inhabitants
2007 B2	Suburb school of a big city 61,500 inhabitants
2007 B3	Suburb school of a big city 19,000 inhabitants
2007 B4	Suburb school of a big city 19,000 inhabitants
2007 B5	Suburb school of a big city 19,000 inhabitants
2007 B6	Suburb school of a big city 19,000 inhabitants
2007 C1	Small urban school in the south of Sweden: 26,400 inhabitants
2007 C2	Small urban school in the south of Sweden: 26,400 inhabitants
2007 D1	Rural school in the north of Sweden: 6500 inhabitants
2007 D2	Rural school in the north of Sweden: 6500 inhabitants
2007 D3	Rural school in the middle of Sweden: 6500 inhabitants
2007 D4	Rural school in the middle of Sweden: 6500 inhabitants
**2016**	Place and school
**48 participants**	
**8 focus group**	
5–8 students/group	
2016 A1	Big city (1 million inhabitants) city centre school
2016 A2	Big city (1 million inhabitants) city centre school
2016 B1	Suburb school of a big city 25,000 inhabitants
2016 B2	Suburb school of a big city 25,000 inhabitants
2016 C1	Small urban school in the south of Sweden: 27,300 inhabitants
2016 C2	Small urban school in the south of Sweden: 27,300 inhabitants
2016 D1	Rural school in the north of Sweden: 6000 inhabitants
2016 D2	Rural school in the north of Sweden: 6000 inhabitants
